# The Significance Application of Indigenous Phytohemagglutinin (PHA) Mitogen on Metaphase and Cell Culture Procedure

**Published:** 2011

**Authors:** Abolfazl Movafagh, Hassan Heydary, Seyed AbdolReza Mortazavi-Tabatabaei, Eznollah Azargashb

**Affiliations:** a *Department of Medical Genetics, Shahid Beheshti University of Medical Sciences, Tehran, Iran.*; b *Department of Medical Anatomy, Shahid Beheshti University of Medical Sciences. Tehran, Iran.*; c *Proteomics Research Center, Facalty of School of Paramedical Sciences, Shahid Beheshti University of Medical Sciences, Tehran, Iran.*; d *Department of Medical Health Community, Shahid Beheshti University of Medical Sciences, Tehran, Iran.*

**Keywords:** Application, Cell culture, Indigenous, Mitotic index, Phytohemagglutinin (PHA)

## Abstract

Phytohemagglutinin (PHA) is a lectin, obtained from the red kidney bean that binds to the membranes of T-cells and stimulates metabolic activity, cell division, *etc. *The object of this research was the comparison between self made PHA (Indigenous) and imported commercial one, following conventional and High Resolution Cell Synchronization technique (HRCS) .From each blood sample of healthy individual donor replicate cell culture with two different PHA (self-made and commercial imported) with same concentration were cultured simultaneously. For culture cells, 3-5 × 106^6^ cells were cultured in 4 mL medium( RPMI 1640 supplemented with 15 per cent heat inactivated fetal bovine serum, 0.1 mL Phytohemagglutinin was added and kept at 37°C in an atmosphere containing 5% CO_2_. The processing of mitotic division from 48 h and 72 h cultures was performed according to the standard and High Resolution Cell Synchronization technique. Cytogenetic studies were performed in 100 normal healthy blood donor individuals. Statistical analysis was performed by SPSS (version 16, Inc.USA) software.Our results indicate that the preparation of fresh Phytohemagglutinin at the time of cell division and cell culture procedure reveals satisfactory score. The overall frequency of mitotic index in our study was better when compared with commercial imported Phytohemagglutinin (p < 0.001).The significant differences in the results may be due to fresh preparation. However, cost effective, easy and nearest approach of this indigenous product and high demand for this product among health care services can be considered.

## Introduction

Phytohemagglutinin (PHA), the lectin extract from the red kidney bean (Phaseolus Vulgaris), contains potent, cell agglutinating and mitogenic activities (1). The subunits of PHA are of two different types, designated leucocyte reactive (L) and erythrocyte reactive (E). L has a high affinity for lymphocyte surface receptors but little for those of erythrocytes and is responsible for the mitogenic properties of the isolectins. The E is responsible for the erythrocyte agglutinating properties. Phytohemagglutinin-P is the protein form and PHA-M is the mucoprotein form of these isolectin (2).

**Figure 1:**
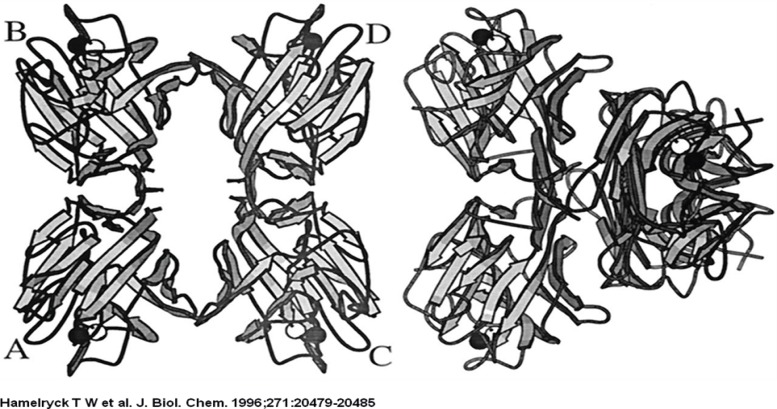
Comparison between ConA and PHA-L

**Figure 2:**
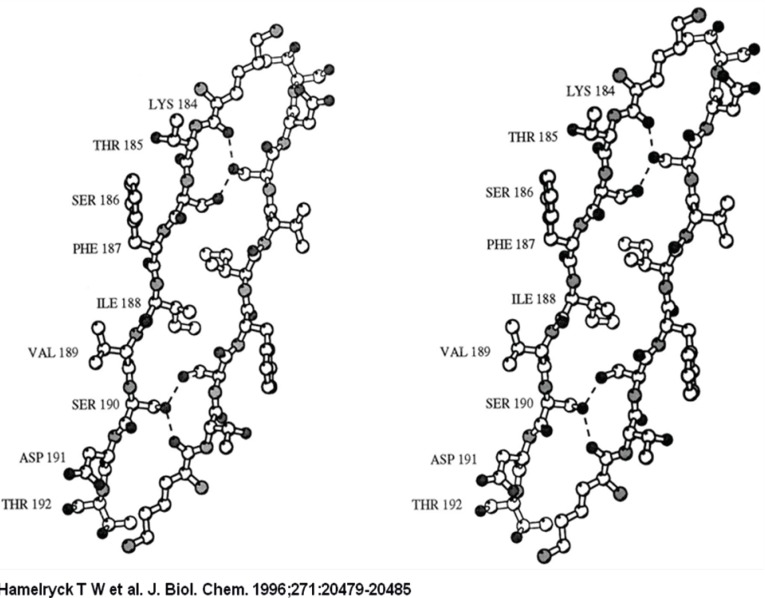
A stereo figure of the dimer-dimer interface in the SBA tetramer

**Figure 3:**
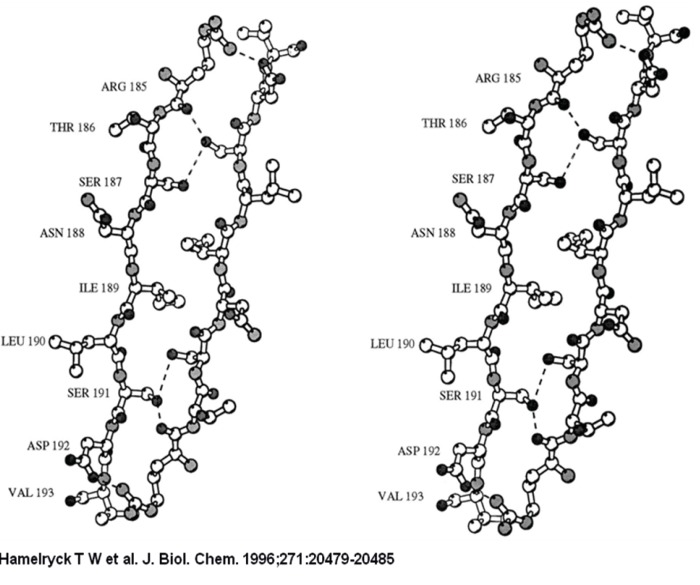
A stereo figure of the dimer-dimer interface in the SBA tetramer

**Figure 4:**
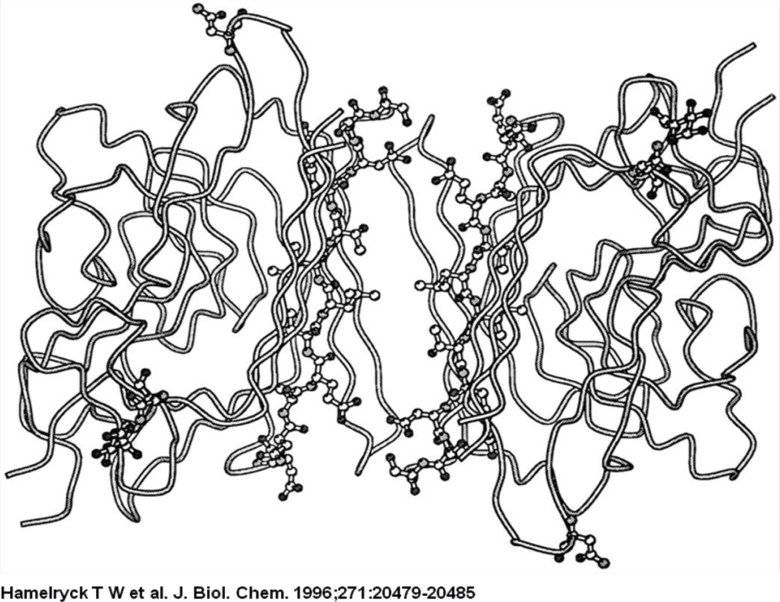
A view from the center of the molecule to the dimer-dimer interface on the putative adenine binding site between dimers A and C

**Figure 5:**
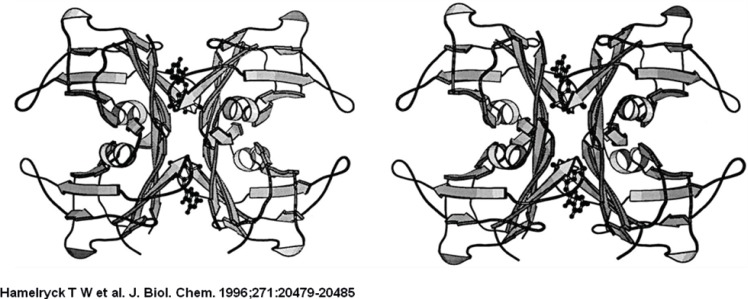
Stereo figure of the transthyretin tetramer complex with 3′,5′-dibromo-2′,4,4′,6-tetrahydroxyaurone, a flavone derivate

**Figure 6:**
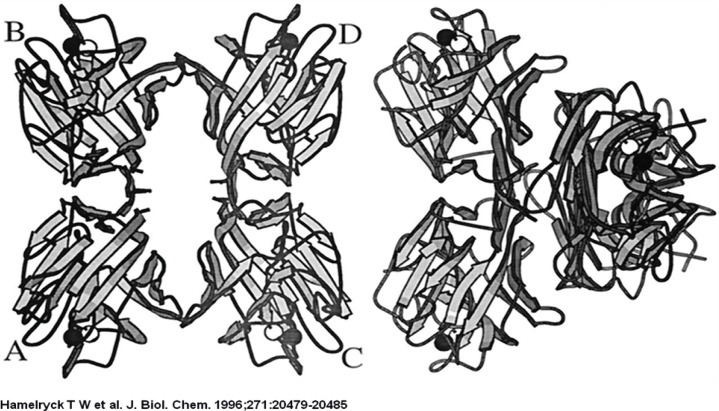
Comparison between ConA and PHA-L

It is assumed that there exist some individual differences in response to PHA and the limitation of only T-lymphocyte stimulation by PHA (3). Therefore, Pokeweed mitogen (PWM) which was known to stimulate T and B-lymphocytes, and some other mitogens such as Concavalin A(Con-A), lipopolysaccharide (LPS), Wheat Germ Agglutinin (WGA) and Soybean Agglutinin (SBA) were used and compared on their mitotic stimulating effects in single use and also combined use of these mitogens (Figures 1-6) (4 ). One of the mitogens, phytohemagglutinin (PHA), has been widely used for the purpose of mitotic stimulation to human lymphocytes, and several different types of PHA, such as PHA-P, M, W and others were compared on their ability to induce mitoses and presented by other workers (5-10). In addition to the growing knowledge of lectins is mammalian lectin is dectin-1, *α β*-glucan receptor, is identified by Gordon and Brown (11). A new type of plant root lectin is found in different leguminous plants but not in plants of different family (12).

Phytohemagglutinin has the potential to induce closer contacts between adjacent cell membranes; it is an *N- *acetylgalactosamine/galactose sugar-specific lectin with wide variety of biological activities (13). Phytohemagglutinin has been successfully used for membrane-induced fusion in human oocytes (14), bovine oocyt (15), and caprine oocytes (16). It was the first direct evidence for the involvement of bacterial lectins in the initiation of infection, the basis for the present attempts in academia and industry to apply carbohydrates for anti-adhesion therapy of such diseases reviewed by Mulvey and *et al *(17).

Since the development of convenient methods of culturing peripheral blood leukocytes (18) in the early 1960s, the study of human chromosomes has been a rapidly advancing, exciting field. Earlier attempt was made to analyze human and other mammalian chromosomes utilized tissues, such as bone marrow and testicular tissue, in which cells in division were present. These investigators tried to adapt plant chromosome methodology to the study of human tissues but were largely unsuccessful. Frustration occurred due to the large number of chromosomes in the human complement, meaning they could not be spread out and separated enough to visualize the entire complement of one cell. For the automation of human chromosome analyses, the accumulation of enormous number of mitoses is thought to be a great advantage (19-29).

In addition to the well established effect of PHA on mitotic stimulation, and also based on the current literature, the advantages of PHA agent would indicate the potential sources for developing novel pharmaceutical preparation. However, reports on the culture condition of human lymphocytes including the stimulating condition by mitogens was examined in detail, were quite limited; likewise, the purpose of the present study is to establish the most appropriate condition in mitotic stimulation to human lymphocytes.

The PHA-L tetramer is shown on the left, the ConA tetramer is shown on the right. The left dimers in both tetramers have the same orientation to emphasize the difference in dimer-dimer packing between PHA-L and ConA. The central channel running between the two dimers in PHA-L is clearly visible.

The 2-fold axis is approximately positioned in the center of the Figure. As in PHA-L, the side chains of 2 Ser (Ser-191 and −187) and 1 Ile residue (Ile-189) intercalate. The main difference with PHA-L is the substitution of Lys-184 in PHA-L by Arg-185 in SBA. The Arg-185 is involved in an additional hydrogen bond with Asp-192. The six hydrogen bonds (Arg-185A O-Ser-191C OG, Arg-185A NH1-Asp-192C OD1, Ser-187A OG-Ser-191C OG, Ser-191A OG-Arg-185C O, Ser-191A OG-Ser-187C OG, Asp-192A OD1-Arg-185C NH1) across the interface are visualized as dashed lines.

The strand that contains the photo affinity labelled residues in both monomers is shown as a ball-and-stick representation. The two glycosylated residues per monomer (Asn-12 and Asn-60) are also shown in ball-and-stick representation, together with the GlcNAc residue bound to Asn-12.

The ligand is bound in the central hole that runs through the molecule, through interactions with side chains of the residues that make up the flanking *β*-strands (mainly Ser, Thr, Ala, and Leu). The binding site possesses 2-fold symmetry. The central hole is approximately 10 Å wide.

The PHA-L tetramer is shown on the left, the ConA tetramer is shown on the right. The left dimers in both tetramers have the same orientation to emphasize the difference in dimer-dimer packing between PHA-L and ConA. The central channel running between the two dimers in PHA-L is clearly visible.

## Experimental

We studied 100 either sex adult normal in order to determine simultaneous effect of two different mitogens for cell division and karyotype procedure. Of them 62 were males and 38 were females. The age of these normal control-blood donors at the time of cell division ranges from 19 to 65 years old. The experiment was evaluated at Tehran during the year 2004-2010. The samples were taken from the normal individuals with their consent. Their personal information was kept confidential. No medicines or drugs were taken by this group for at least one month prior to sampling. Also, those suffering from genetic disorders, cancers, chronic and acute leukemia, syndromes, current viral infections, general or dental X-rays more than a month were excluded from the study; they also had no recorded overexposure in their personnel medical documents.

*Cytogenetics*

In each sample, 0.5-1.0 mL Peripheral blood (PB) was obtained from each individual. For culture purposes, 3-5 × 10^6^ cells were done so in 4 mL medium (RPMI 1640, Gibco-BRL Grand Island, NY, USA) supplemented with 20 per cent heat inactivated fetal bovine serum (Gibco-BRL Grand Island, NY, USA) and the total preparation of Phytohaemagglutinin (5 μL/mL according to the manufacture description), also 5 μL/mL Phytohaemagglutinin with similar concentration was prepared by own in research laboratory at 37°C in an atmosphere containing 5% CO_2_. Briefly, the cultured cells were then treated with Colcemid (Gibco-BRL Grand Island, NY, USA) final concentration, 10 μg/mL and incubated at 37°C for an additional 20-60 min. The contents of the tube were then centrifuged for 10 min at 1000 rpm, re-suspended in 10 mL of 75 mM KCl (0.56%), and pre-warmed to 37°C for 20 min. At this stage, 1 mL of Carnoys Fixative (3:1 methanol: acetic acid) was added in to the tube, and this fixation step was repeated four times. Ten slides were prepared for each culture and stained for 3 min with Giemsa. Slides were examined with an Olympus model BH-2 light microscope. Experimental examinations of mitotic division were described according to ISCN (30).

*Statistical analysis*

The data of MI on slides were pooled after performing the t-test and the statistical differences yielding p < 0.05 were considered significant. The data of mitotic index (MI) analysis was performed by SPSS (version 16, Inc.USA) software.

## Results and Discussion

This series of 100 normal individual blood donors could be grouped into two experiments (A and B) replicate categories according to the nature of two different mitogen cell division treatments. Two different mitogens viz, self made indicated as Exp-A and commercial imported designate as Exp-B were used simultaneously for cell division.

**Figure 7:**
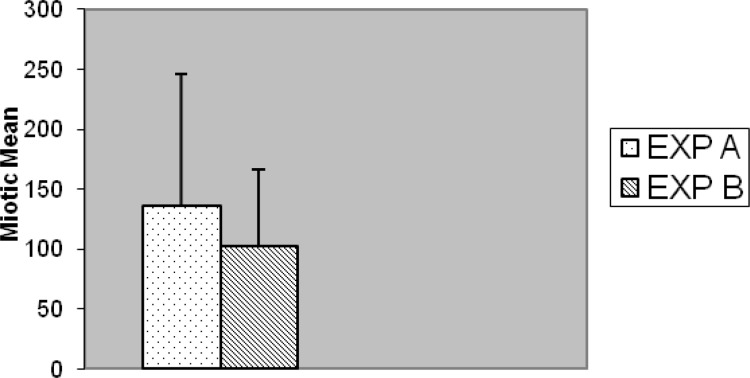
Comparison as a whole of mitotic index between EXP A and EXP B (p < 0.001).

Slides for either of the experiments were coded and randomized prior to scoring for MI. Consecutive metaphases on blind selected slides per replicated preparation were scored whether or not; they were suitable for full analysis. Well metaphases spread on two to five slides from each of two experiments cell culture (replicate) with different stimulated cell division were scored. In five cases no outgrowth was obtained, hence substituted with another five normal blood sample (cases) was prepared with fresh simultaneous mitogens treatment. Generally, chromosome preparation obtained from Exp-A exhibited higher mitotic activity than Exp-B individual culture preparation of commercial mitogen material as shown in Figure 7. Almost, no consistent differences for MI were observed between Exp-A and Exp-B for 8 (8%) cases. Proportions of mitotic indicex revealed according to the treatment of both experiments are summarized in Figure 7. Seventy-three percent (73%) of the preparation was harvested after 3 days (72 h) and the remaining twenty seven (27%) cell culture preparation arrested at 48 h. This application of alternative treatment has been followed by standard and conventional method in accordance with ISCN for firm conclusion.

Taking into account all the results, some differences were found between two experiments, notably, in Exp-A, 9 (9%) individuals had > 300 MI; 6 (6%) individuals had > 200 < 300 MI; 8 (8%) individuals had > 100 < 200 MI; 26 (26%) individuals had < 100 MI.

In Exp-B; no (0%) individuals had > 300 MI; 10(10%) individuals had > 200 < 300 MI; 12 (12%) individuals had > 100 < 200 MI; 27 (27%) individuals had < 100 MI.

**Figure 8:**
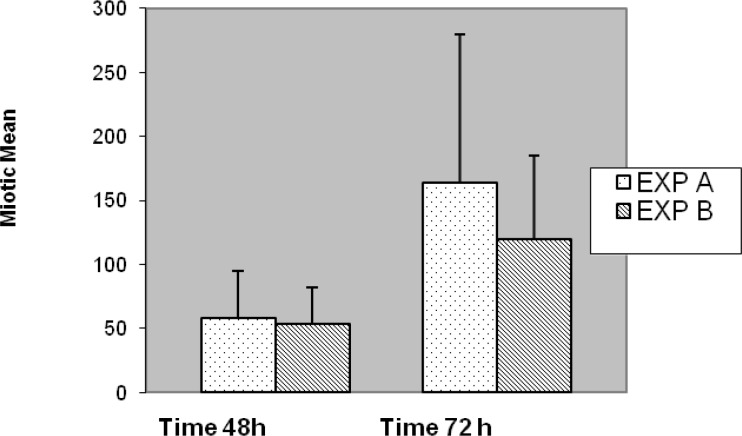
Comparison of mitotic index for both Exp A and B together with two different culture period (p < 0.001

**Figure 9:**
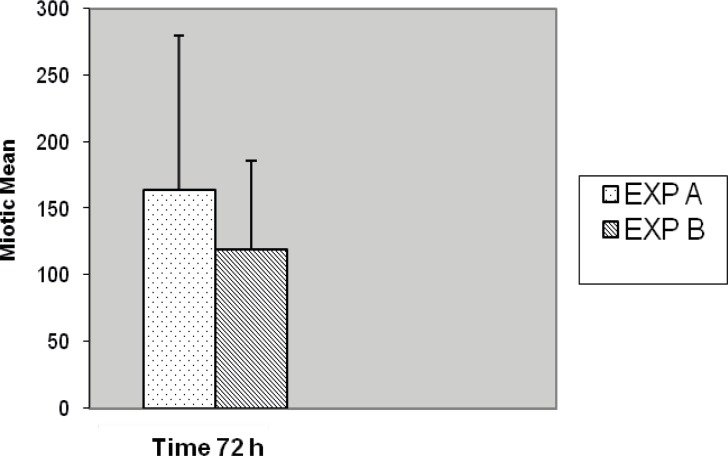
Comparison of mitotic index between EXP A and EXP B with 72 h culture period (p < 0.001).

**Figure 10:**
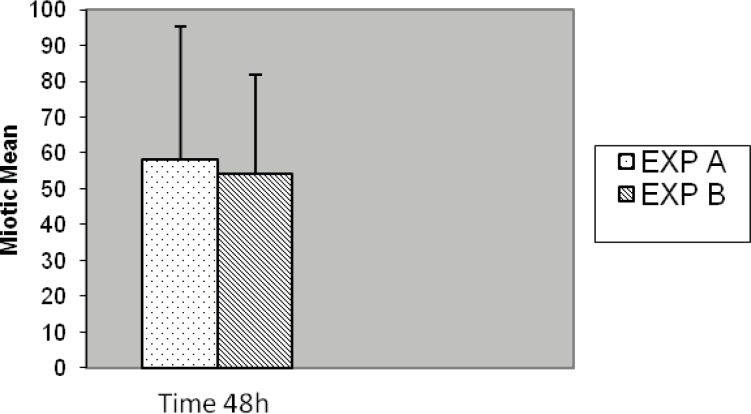
Comparison of mitotic index between EXP A and exp B with 48 h culture period (p = 0.510).

The results of Exp-A, were as follows: the mean value of the MI is significantly greater, 135.77, whereas in the Exp-B it is 102.16 (t = 5.178, df = 99, p < 0.001) (Figure 7). The differences were significantly increased during the 72 h of application with respect to 48 h for both Exp–A and B (p < 0.001) (Figure 8). However, the differences were not significant for distribution of gender between two experiments (A and B), (p < 0.007). The differences were also significant between Exp A and Exp B at 72 h (p < 0.001) (Figure 9). The frequency of Exp-A and Exp-B did not show any significant difference (p = 0.510) at 48 h (Figure 10).

Many line of evidence have demonstrated the role of PHA with wide variety of biological activities such as mitogen stimulation of lymphocytes and agglutination of cancer cells, the role of various glycosyltransferase in the pathogenicity of cancer cells (30), fusion of mammary gland epithelial cells into enucleated oocytes in caprine, bovine, goats (14-16). Lectin precipitation using Phytohemagglutinin are considered as a candidate biomarkers for colorectal cancer discovery (31). PHA skin test is an easy and a reliable method to affect the immune response of surgical patients; it has many advantages above the commonly employed primary and secondary antigens (32). Insulin, like growth factor-1, promotes cord blood T-cell mutation and inhibits its spontaneous and Phytohemagglutinin induced apoptosis through the down regulation of Fas expression (33).

The present work confirms and extends our previous findings that most normal control cases revealed well spread MI when self-made mitogenic product were used as a normal control with comparison of mitotic index in malignant diseases (34). 

Cytogenetic findings in hematologic malignancies are useful for their diagnosis and prognostic value. Generally, those samples with low cell counts fail to produce sufficient metaphases for accurate diagnosis. Even when cultures are successful, the chromosomes are often highly contracted, and the metaphases spread can be of low quality (24). On the basis of present investigation significant differences (p < 0.001) between Exp A and B for MI were found through applying simultaneous treatment of both mitogen reagents. It is reasonable to state that this variation is a sufficiently significant reason to use application of indigenous product than imported commercial PHA mitogen. Moreover, with the improved MI obtained from this procedure presented here, a more precise identification of chromosomal abnormalities in routine laboratory diagnosis and neoplasia is possible.

Furthermore, it is sensible to state that lectins are believed to act as modulations of cell substratum interactions and to be essential for the normal differentiation and growth of all multicellular animals. They are capable of inducing cell proliferation, cell arrest, or apoptosis (physiological cell death) and have been implicated in organ morphogenesis, tumor cell metastasis, leukocyte trafficking, skin test for immunological assessment response, and inflammation, as well as recognition of extracellular matrix (3).

Our attempts to develop a self prepared PHA have led us to the following conclusions. The fresh preparation of indigenous PHA mitogen we have used to identify chromosome preparation for normal as well as other malignancies are superior to other imported commercial product due to indigenous, more metaphases scores, freshness, cost effective, easy and accessibility. Briefly, in our country the challenge for the biotechnology and therapeutic product as a profession is in initial stages compared to the developing countries, although the results of this study has revealed a high demand for this product among health care and clinical dimensions. However, additional experiments are necessary to establish the most appropriate condition in use of different mitogens for metaphase and cell stimulating preparation.

## References

[1] Goossens A, Geremia R, Bauw G, Van Montagu M (1994). Angenon GIsolation and characterisation of arcelin-5 proteins and cDNAs. Eur. J. Biochem.

[2] Thomas W, Hamelryck, Minh H D T, Poortmans F, Maarten J, Chrispeels, Lode W, Remy L (1996). The crystallographic Structure of phytohemagglutinin-L. J. Biol. Chem.

[3] Sharon N, Lis H (2004). History of lectins: from hemagglutinins to biological recognition molecules. Glycobiology.

[4] Sofuni T, Yoshida M (1992). Combined use of several mitogens for mitotic stimulation to human lymphocytes. J. Radiate. Res.

[5] Morgan WT, Watkins WM (2000). Unravelling the biochemical basis of blood group ABO and Lewis antigenic specifity. Glycon. J.

[6] Onaga S, Taira T (2008). A new type of plant chitinase containing LysM domains from afern (Pteris ryukyuensis): roles of LysM domains in chitin binding and antifungal activity. Glycobiology.

[7] Velloso LM, Svensson K, Schneider G, Pettersson RF, Lindqvist Y (2002). Crystal structure of the carbohydrate recognition domain of p58/ERGIC-53, a protein involved in glycoprotein export from the endoplasmic reticulum. J. Biol. Chem.

[8] Meesmann HM, Fehr EM, Kierschke S, Herrmann M, Bilyy R, Heyder P, Blank N, Krienke S, Lorenz HM, Schiller M (2010). Decrease of sialic acid residues as an eat-mesignal on the surface of apoptotic lymphocytes. J. Cell Sci.

[9] Shridhar S, Chattopadhyay D, Yadav G (2009). PLec Dom. a program for identification and analysis of plant lectin domains. Nucleic Acids. Res.

[10] Espinosa EP, Perrigault M, Ward JE, Shumway SE, Allam B (2009). Lectins associated with the feeding organs of the oyster Crassostrea virginica can mediate particle selection. Biol. Bull.

[11] Brown GD, Gordon S (2001). Immune recognition: a new receptor for beta glucans. Nature.

[12] Kalsi G, Etzler ME (2000). Localization of a Nod factor-binding protein in legume roots and factors influencing its distribution and expression. Plant Physiol.

[13] Zhang YL, Liu FJ, Sun DQ, Chen XQ, Zhang Y, Zheng YM, Zhao MT (2008). Phytohemagglutinin improves efficiency of electrofusing mammary gland epithelial cells into oocytes in goats. Theriogenology.

[14] Tesarik J, Nagy ZP, Mendoza C, Greco E (2000). Chemically and mechanically induced membrane fusion: non-activating methods for nuclear transfer in mature human oocytes. Hum. Reprod.

[15] Hongs SB, Uhm SJ, Park CY, Gupta MK, Chung BH, Chug KS, Lee HT (2005). Developmental ability of bovine embryos nuclear transferred with frozen-thawed or cooled donor cells. Asian Australian J. Animal Sci.

[16] Begin I, Bhatia B, Rao K, Keyston R, Pierson JT, Neveu N (2004). Pregnancies in the presence of lectin. Report Fertil. Dev.

[17] Mulvey G, Kitov P, Marcato P, Bundle DR, Armstrong GD (2001). Glycan mimicry as a basis for novel anti-infective drugs. Biochimie.

[18] Nowell PC, Hungerford DA (1960). Chromosome studies on normal and leukemic human leukocytes. J. Natl. Cancer Inst.

[19] Ikehara S (2009). A new bone marrow transplantation method for stem cell disorders. Ann. NY Acad. Sci.

[20] Ghaffari MR, Kadkhodaei-Elyaderani M, Saffari MR, Pedram M (2010). Monitoring of serum nitric oxide in patients with acute leukemia. Iranian J. Pharm. Res.

[21] Tsao GJ, Allen JA, Logronio KA, Lazzeroni LC, Shizuru JA (2009). Purified hematopoietic stem cell allografts reconstitute immunity superior to bone marrow. Proc. Natl. Acad. Sci.

[22] Horwitz ME (2007). Sources of human and murine hematopoietic stem cells. Curr. Protoc. Immunol.

[23] Yunis JJ (1981). New chromosome techniques in the study of human neoplasia. Human Pathol.

[24] Misawa S, Horiike S, Taniwaki M (1988). Detection of karyotypic abnormalities in most patients with APL by adding ethidium bromide to short term culture. Leuk. Res.

[25] Kushida T, Inaba M, Ikebukuro K, Ngahama T, Oyaizu H, Lee S, Ito T, Ichioka N, Hisha H, Sugiura K, Miyashima S, Ageyama N, Ono F, Iida H, Ogawa R, Ikehara S (2000). A new method for bone marrow cell harvesting. Stem Cell.

[26] Yazdanparast Y, Moosavi MA (2004). Gnidilatimonoein from Daphne mucronata induced differentiation and apoptosis in leukemia cell lines. Iranian J. Pharm. Res.

[27] Ikehara S (2009). A new bone marrow transplantation method for stem cell disorders. Ann. NY Acad. Sci.

[28] Tsao GJ, Allen JA, Logronio KA, Lazzeroni LC, Shizuru JA (2009). Purified hematopoietic stem cell allografts reconstitute immunity superior to bone marrow. Proc. Natl. Acad. Sci. USA.

[29] Wright DE, Cheshier SH, Wagers AJ, Randall TD, Christensen JL, Weissman IL (2001). Cyclophosphamide/granulocyte colony-stimulating factor causes selective mobilization of bone marrow hematopoietic stem cells into the blood after M phase of the cell cycle. Blood.

[30] Brothman AR, Persons DL, Shaffer LG (2005). Nomenclature evolution. Changes in the ISCN from the.

[31] Kim YS, Son OL, Lee JY, Kim SH, Oh S, Lee YS, Kim CH, Yoo JS, Lee JH, Miyoshi E, Taniguchi N, Hanash SM, Yoo HS, Ko JH (2008). Lectin recipitation using phytohemagglutinin-L (4) coupled to avidin-agarose for serological biomarker discovery in colorectal cancer. Proteomics.

[32] Meijer S, Bom-van Noorloos AA, Visser JJ (1984). Phytohemagglutinin skin test for theimmunological assessment of the surgical patient. Eur. Surg. Res.

[33] Tu W, Cheung PT, Lau YL (2000). Insulin-like growth factor I promotes cord blood T cell maturation and inhibits its spontaneous and phytohemagglutinin-induced apoptosis through different mechanisms. J. Immunology.

[34] Movafagh A, Hajiseyed Javadi M, Arian N, Katosian B (2007). The possible association between constitutive hetochromatin polymorphism and human leukemia. Yakhteh Med. J.

